# Interprofessional Education in Child Protection for Preservice Health and Allied Health Professionals: A Scoping Review

**DOI:** 10.1177/15248380231221279

**Published:** 2024-01-28

**Authors:** Lauren Elizabeth Lines, Tracy Alexis Kakyo, Helen McLaren, Megan Cooper, Nina Sivertsen, Alison Hutton, Lana Zannettino, Rebecca Starrs, Donna Hartz, Shannon Brown, Julian Grant

**Affiliations:** 1 Caring Futures Institute, College of Nursing and Health Sciences, Flinders University, Adelaide, SA, Australia; 2 College of Education, Psychology, and Social Work, Flinders University, Adelaide, SA, Australia; 3 Faculty of Health Sciences, Sámi Nursing, UiT Arctic University of Norway, Hammerfest, Norway; 4 School of Nursing and Midwifery, University of Newcastle, Callaghan, NSW, Australia; 5 College of Nursing and Health Sciences, Flinders University, Adelaide, SA, Australia; 6 School of Nursing and Midwifery, Western Sydney University (Parramatta & South) and Western Sydney Local Health District, Penrith, NSW, Australia; 7 Molly Wardaguga Research Center, Charles Darwin University, Casuarina, NT, Australia; 8 College and Research Services, Flinders University, Adelaide, SA, Australia; 9 Faculty of Science and Health, Charles Sturt University, Bathurst, NSW, Australia

**Keywords:** interprofessional education, child protection, health professional, allied health professional, scoping review

## Abstract

Health and allied health professionals are uniquely positioned to collaborate in prevention, early intervention and responses to child maltreatment. Effective collaboration requires comprehensive interprofessional education (IPE), and inadequate collaboration across sectors and professions continually contributes to poor outcomes for children. Little is known about what interprofessional preparation health and allied health professionals receive before initial qualification (preservice) that equips them for interprofessional collaboration and provision of culturally safe care in child protection. This scoping review aimed to identify what is known internationally about IPE in child protection for preservice health and allied health professionals. Thirteen manuscripts reporting 12 studies met the inclusion criteria and were included in the synthesis. Key characteristics of the educational interventions are presented, including target disciplines, core content and their learning objectives and activities. Findings demonstrated primarily low-quality methodologies and educational interventions that had not been replicated beyond their initial context. Many educational interventions did not provide comprehensive content covering the spectrum of prevention, early intervention and responses for all types of child maltreatment, and/or did not clearly indicate how IPE was achieved. Key challenges to delivering comprehensive interprofessional child protection include lack of institutional support and competing priorities across disciplines who must meet requirements of separate regulatory bodies. Consequently, there is a need for further development and robust evaluation of educational interventions to explore how interprofessional collaborative skills for child protection can be developed and delivered in preservice health and allied health professional education.

## Introduction

There is global recognition that child maltreatment is a serious problem. Globally, around 1 billion children experience violence each year (Hillis et al., 2016; World Health Organization, 2022c). According to the World Health Organization (2022a), child maltreatment includes:

. . .all types of physical and/or emotional ill-treatment, sexual abuse, neglect, negligence and commercial or other exploitation, which results in actual or potential harm to the child’s health, survival, development or dignity in the context of a relationship of responsibility, trust or power.

Circumstances that place children at risk of maltreatment include modifiable factors like poverty, domestic and family violence, social exclusion, lack of access to culturally safe services and intergenerational trauma (Featherstone et al., 2017; Higgins et al., 2019). These factors associated with increased risk for children are exacerbated by global events, including climate change, increasing socioeconomic inequities and ongoing impacts of the Covid 19 pandemic (World Health Organization, 2020). Child maltreatment has severe and lasting impacts upon children (Widom, 2022), with children impacted by maltreatment experiencing poorer lifelong physical and psychological health, lower educational attainment and increased socioeconomic disadvantage which is transmitted intergenerationally (Vizard et al., 2022; World Health Organization, 2022c). Child maltreatment also has broader socioeconomic consequences stemming from the impacts of psychological distress that increase costs of health and social care, criminal justice responses, and loss of productivity (Conti et al., 2021; Fang et al., 2015). Consequently, effective and culturally safe prevention, early intervention and response are key to enable children to thrive and reach their full potential (World Health Organization, 2006).

Children and families encounter a range of professionals during the early years, including health, welfare, and other allied health professionals (Russ et al., 2022; World Health Organization, 2020). Key disciplines include occupational therapy, physiotherapy, speech pathology, medical professionals, psychologists, midwives, nurses, and social workers (Grant et al., 2016). In many countries, these health and welfare professionals are mandated reporters of child maltreatment and are aware of their obligations in keeping children safe and reporting suspicions of child maltreatment (McLaren, 2007). Accordingly, these professionals are well-placed to intervene early to support families experiencing adversities to mitigate risk of abuse and neglect or report abuse when required (Lonne et al., 2013; Sedlak et al., 2022). When children and families experience adversities, they may have complex health, wellbeing, developmental, cultural, and safety needs. Supporting often means navigating wicked social problems across system levels, and this is best approached using a collaborative response (Higgins et al., 2022) inclusive of the child’s community (Lonne et al., 2020). One key challenge is that service responses are often fragmented and siloed, leading to missed opportunities for early intervention or identification of child maltreatment, lack of strategic communications, and unnecessary duplication of services (Higgins et al., 2019). These collaborative challenges can also add complexity to professionals’ experiences of mandatory reporting, such as when negotiating with key stakeholders (colleagues, families, and child protection services) which highlights different interprofessional perspectives and power imbalances (Kuruppu et al., 2023; McTavish et al., 2017).

There are many well-known cases where professional silos, poor communication, and uncoordinated responses have led to some of the worst outcomes for children (Parton, 2004), and in particular First Nations children (Government of Canada, 2020; Lonne et al., 2020). The links between poor coordination and unnecessary child deaths are so prevalent that many countries have legislated child death review processes (Raman et al., 2017). Despite legislated processes, children continue to die from maltreatment where inadequate information sharing and poor coordination are contributing factors. At an individual professional level, there are many barriers to interprofessional collaboration, including disciplines that are inadequately educated, equipped, and supported for interprofessional practice (Bogossian & Craven, 2021; Grant et al., 2016; Sivertsen et al., 2020). Effective interprofessional education (IPE) is the key to developing a workforce capable of breaking down practice silos, with the intent of keeping children safe.

Effective interprofessional practice is increasingly recognized as an essential component of comprehensive, high-quality care (Reeves et al., 2016). IPE in preservice education can provide essential foundations for collaborations that are core to early identification of risk factors and the prevention of child maltreatment (Reeves et al., 2016). IPE needs to occur at the preservice level (prior to initial qualification) so health and allied health professionals graduate with the knowledge and requisite skills for interprofessional practice (Rogers et al., 2017; World Health Organization, 2010). According to the World Health Organization (2010, p. 7), IPE occurs “*when students from two or more professions learn about, from and with each other to enable effective collaboration and improve health outcomes*.” Existing research has reported on preservice education separately in the areas of (1) IPE or (2) child maltreatment (Thistlethwaite & Moran, 2010; Walsh et al., 2022). However, the nature and scope of IPE for preservice education in the complex area of child maltreatment for health and allied health professionals is not known. This scoping review therefore aimed to identify what is known about IPE in child maltreatment for preservice health and allied health professionals to identify gaps in existing literature and the need for future research.

## Methods

A scoping review methodology was chosen because this area of research has not been comprehensively reviewed and we wanted to map “the breadth and depth” (Levac et al., 2010) of published work without limiting results to a specific study design (Peters et al., 2015). This scoping review followed Arksey and O’Malley’s (2005) framework, and recent methodological updates (Levac et al., 2010; Peters et al., 2015). The stages of this scoping review were (1) Identifying the research question, (2) Identifying relevant studies, (3) Selecting studies, (4) Charting the data, and (5) Collating, summarizing and reporting results (Arksey & O’Malley, 2005). The review was conducted based on a protocol that was registered in OSF (doi: 10.17605/OSF.IO/K5CN7) prior to the study (Lines 2024). The reporting of this review adhered to the guidelines provided by the Preferred Reporting Items for Systematic reviews and Meta-analyses extension for Scoping Reviews (PRISMA-ScR) (Tricco et al., 2018).

### Identifying the Research Question

The research question guiding this review was: What is known about IPE in child protection for preservice health and allied health professionals? Child protection was defined broadly to incorporate all actions across the spectrum of prevention, early support and responses to maltreatment, and incorporated all forms of child maltreatment as defined by the World Health Organization (2022a).

### Identifying Relevant Studies

The search strategy for this review was broad. It aimed to identify both published and unpublished academic research, using a three-step approach. A preliminary search was conducted in Medline (via Ovid) and CINAHL (via EBSCO) to identify relevant articles, keywords, and scope. The identified keywords and index terms guided the development of a comprehensive search strategy in Medline (see Supplemental Online 1). Key words included combinations of all variations of the following words: “interdisciplinary,” “multidisciplinary,” “interprofessional,” “care team,” “child abuse,” “child maltreatment,” “child neglect,” “safeguarding,” “child welfare,” “domestic violence,” and “sexual abuse.” The retrieved articles from the Medline search were assessed to ensure the inclusion of key publications. The search strategy, including keywords and relevant index terms, was adapted for other bibliographic databases by a research librarian (SB), including PsycINFO (via Ovid SP), Cumulated Index to Nursing and Allied Health Literature CINAHL (EBSCOhost), Web of Science (Clarivate Analytics), Scopus (Elsevier), ProQuest Central, and ProQuest Social Science Premium Collection (Clarivate). ProQuest Dissertations & Theses was searched for unpublished material. Each database search strategy was run on February 8, 2023. Additionally, a supplemental search was conducted using Google Scholar and hand searching of key journals in interprofessional care or child maltreatment to identify studies meeting the inclusion criteria. The search encompassed articles from the inception of each database until 2023, with no restriction on the publication date of the articles meeting the keyword inclusion criteria.

To further expand the search, the reference lists of all included sources were screened for additional studies. In addition, the 12 citations were entered as seed papers into a free online scholarly publication discovery and mapping tool, known as ResearchRabbit (Chandra et al., 2021). This Artificial Intelligence (AI) tool is designed to support unstructured searching via data mining of publicly available scholarly papers and information relevant to seed papers uploaded (Cole & Boutet, 2023). The use of this tool served two purposes: 1. A final forward/backward check for any items which may have been missed during systematic and subsequent hand searching; and 2. A discovery of timeline trends for the studies included and similar works. No additional relevant studies were identified.

### Selecting Studies

The inclusion and exclusion criteria were discussed by the research team prior to screening by titles and abstracts. To be included, studies needed to focus on education or training for interprofessional practice in child protection for health and allied health students that was undertaken during the course of their studies leading to initial qualification. Studies were eligible for inclusion if they included two or more disciplines from health or allied health (World Health Organization, 2010) with at least one cohort of preservice students. Studies were excluded if the education or training did not focus on interprofessional practice in child protection or was provided exclusively to a cohort of professionals who already had achieved their initial qualification.

**Figure 1.**
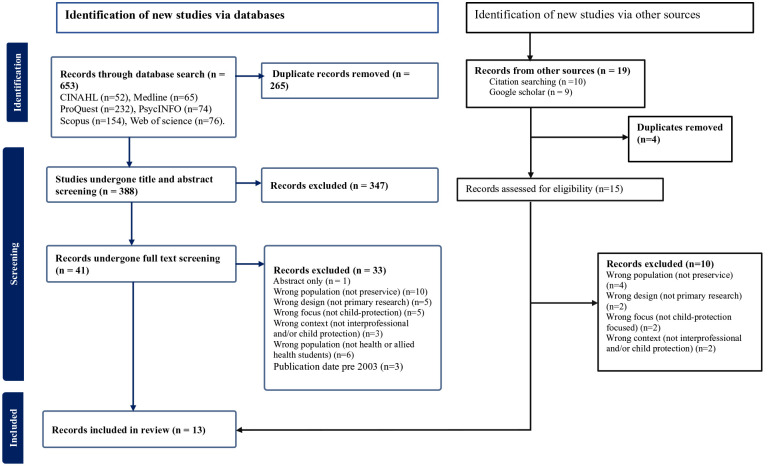
PRISMA flow chart.

Following the search, the 672 citations identified were collated and uploaded into Covidence and 269 duplicates removed. Following pilot testing, 401 articles underwent title and abstract screening by three reviewers (LL, TK, RS). The relevant studies were retrieved in full, and their citation details imported into Covidence. The full text of 55 citations was assessed in detail against the inclusion criteria by four independent reviewers (LL, TK, NS, MC). A manual search in Google Scholar and a thorough examination of the reference lists of the articles that met the inclusion criteria retrieved a further 19 articles which were also screened for eligibility. Reasons for exclusion of papers at full text that did not meet the inclusion criteria were recorded as shown in Figure 1 (PRISMA chart). Any disagreements that arose between the reviewers at each stage of the selection process were resolved through discussion or with an additional reviewer. At the end of the selection process, 17 full text articles covering 15 educational interventions were identified. However, following team discussions of relevance to contemporary child protection policy and practice, articles more than 20 years old (pre 2003) were excluded from analysis. Thus 13 manuscripts representing 12 educational interventions were considered in the subsequent stages of the review process.

### Charting the Data

Two reviewers (LL & TK) independently assessed each article that met the inclusion criteria using a data extraction tool designed by the authors, pilot tested and imported into Covidence for the process of extraction. Information extracted by the two reviewers (LL & TK) included specific details about the study which were: author names, date of publication, title of publication, study setting, study design, data collection methods, and sample characteristics. Also extracted was information about the education intervention: name of the intervention, underpinning theories, development of the intervention, course aims, description of the participants, course structure, mode of delivery, assessment methods, plus the evaluation methods, tools, and outcomes. Any disagreements during data extraction were resolved in a discussion between the reviewers to achieve consensus. Key themes and patterns were collaboratively identified by the two reviewers (LL & TK) to present a descriptive overview of key content and approaches within each educational intervention aligned with the review aim. Key themes and patterns were discussed and further developed and refined in consultation with the authorship team.

### Collating, Summarizing, and Reporting the Results

The included articles were analyzed through basic descriptive summaries using a template that was developed and pilot tested by two authors (LL & TK). Analysis was informed by the research question that guided this scoping review. Quality of evidence was not assessed as the aim of this scoping review was to present an overview of existing evidence irrespective of quality (Peters et al., 2015).

## Results

This scoping review aimed to identify what is known about IPE in child protection for preservice health and allied health professionals.

### Study Characteristics

Thirteen manuscripts reporting 12 studies published within the past 20 years (2003–2023) were included in synthesis; a summary of key characteristics is presented in Table 1. Studies reported interprofessional educational interventions for preservice health and allied health students in six countries (USA *n* = 5, Australia *n* = 2, Germany *n* = 2, Canada *n* = 1, Norway *n* = 1, Turkey *n* = 1). A wide range of health and allied health disciplines were represented; disciplines represented more than once were nursing (*n* = 8), medicine (*n* = 5), social work (*n* = 6), occupational therapy (*n* = 4), psychology (*n* = 3), physiotherapy (*n* = 3), pharmacy (*n* = 2), radiography (*n* = 2) and dentistry (*n* = 2). In addition to educating health and allied health disciplines, several studies (Almendingen et al., 2021; Dolunayğ-Cuğ et al., 2022; Straub et al., 2017; Stuck et al., 2020; Whiteley et al., 2014) included further disciplines including education (*n* = 5) and law (*n* = 1). One study failed to clearly identify students’ disciplines in their sample (Johnson, 2015), and other studies included pooled samples of preservice and post-graduate students (Johnson et al., 2022; Sidelinger et al., 2005; Straub et al., 2017; Victor-Chmil & Foote, 2016). Two articles described a program that had been developed and implemented, but not formally evaluated and therefore contained limited specific detail (Johnson, 2015; Sidelinger et al., 2005).

**Table 1.** Summary of Included Studies.

First Author, Year and Location	Design/Methods	Program Name and Description	Learning Objective(s)	Conceptual Framework(s)	Duration and Structure	Student Sample (Professions, Year Level)	Outcome Measures
Kuliukas et al. (2016) Perth, Australia	Post-test	Interprofessional day of hi-fi simulation of family and domestic violence (FDV)	- Increase awareness of FDV in hospital settings - Understand interprofessional roles of social work and midwifery - Understand screening tools and risk assessment - Develop interprofessional communication skills - Reflect upon processes to make decisions in context of FDV	IPE (not defined)	Length and pattern of delivery not stated Three components: - Pre-brief - Clinical simulation - Student debriefing	Second year undergraduate social work students (*n* = 7) Midwifery (*n* = 6)	Self-report (Likert and free-text) of realism and satisfaction Focus groups
Almendingen et al. (2021) Oslomet, Norway	Pre/post-test	INTERACT (Interprofessional Interaction with Children and Young People)	Learning objectives not explicit; Intervention designed to meet demands for better coordination of children and young people’s services, interaction between professionals, and cooperation between children/young people and professionals	Bronfenbrenner's system's theory Social constructivism United Nations Convention on the Rights of the Child Norwegian law IPC IPL	Two-day seminar. - Day one: children’s rights - Day two: interprofessional work with children, young people, and families	Undergraduate students - Early childhood education - Primary and lower secondary teacher education - Teacher education in art and design - Physiotherapy - Nursing - Social work - Child welfare - Occupational therapy	Student self-reported knowledge and importance of topic (Likert scale) Facilitator perceptions of impact on students’ knowledge (Likert scale)
Dolunayğ-Cuğ et al. (2022) Ankara, Turkey	Qualitative (phenomenology)	Two-Step Prevention Project launched by Turkish Society for Prevention of Child Abuse and Neglect (TSPCAN)	Train undergraduate students while increasing their awareness of CM	Children’s rights Multidisciplinary approach (not defined)	Two-step project: - Step one: 12 weeks of 2 hourly seminars Step two: 8-weeks practical in primary school	Undergraduate students (*N* = 20): - Law - Social work - Psychology - Education - Psychological counseling - Child development - Medicine - Nursing	Open-ended questionnaire and focus group discussions
Johnson (2015) Georgia, USA	Not stated	Child Advocacy Studies Training (CAST) certificate program	- Understand child maltreatment (CM) - Understand factors leading to CM and effective responses - Understand roles and responsibilities of disciplines working in CM - Understand legal system and CM - Understand importance of mandated reporting and confidentiality - Understand interventions used by different disciplines	Not stated	Proposed structure four courses: - Perspectives of CM and child advocacy - Professional and system responses to child advocacy - Internship in child-related agency - - Elective course (choose one of four topics)	Not stated	Proposed evaluation: change in knowledge, attitudes and beliefs
Johnson et al. (2022) New England, USA	Pre/post-test	Violence across the lifespan (VAL) interprofessional education modules	Each module had specific objectives. Human Trafficking and the Healthcare Professional - Describe the various types of human trafficking - Identify the signs of a client/patient who may be trafficked - Discuss how the interprofessional healthcare team can provide optimal patient-centered care to trafficked persons Child Maltreatment and the Healthcare Professional - Define CM - Recognize warning signs of CM - Discuss how interprofessional healthcare team can identify CM Intimate Partner Violence and the Healthcare Professional - Understand intimate partner violence (IPV), including prevalence, risk factors, cycle of violence, and outcomes - Recognize signs of IPV - Describe screening tools to identify IPV - Discuss how interprofessional healthcare team can assist early recognition of IPV	IPE (not defined) Trauma informed	Three online modules (lectures and discussion forums): - Human Trafficking and the Healthcare Professional - CM and the Healthcare Professional IPV and the Healthcare Professional	*N* = 672 health profession students (graduate and undergraduate): - Dentistry - Emergency medical services - Medicine - Nursing - Occupational therapy - Pharmacy - Physical therapy Public health	Self-reported knowledge (Likert scale) Open-ended reflective questions
Rodriguez-Frau et al. (2005) Puerto Rico	Post-test	Introduction to Violence Prevention in Children and Youth (INTD 4065)	To train undergraduate health professional students in youth violence prevention	Core competencies for youth violence prevention health (Knox et al., 2001)^1^ Social Constructivism	- Introduction to violence and violence prevention - Violence in children and youth: a public health problem - Human development and violence prevention - Effects of violence in children and youth - Models for violence prevention - Community role in violence prevention - Programs in children and youth violence prevention - Health professional roles in violence prevention	*N* = 26 undergraduate students: - Nursing - Dental - Radiology - Physical therapy - Occupational therapy - Medical record technicians	Self-reported satisfaction Open-ended level of satisfaction survey Strengths, weaknesses and recommendations (open-ended)
Sidelinger et al. (2005) Multi-site: California, Hawaii and Puerto Rico	Not described	Youth Violence Prevention program—three variants across study sites	Not stated	Problem-based learning Experiential learning	Implemented over 4 years 2-week experiential rotation at one site; details not described for other sites	Undergraduate and post-graduate students: - Physicians - Nurses - Allied health professional	Process-oriented measures for evaluation (details not provided)
Smith et al. (2005) New South Wales, Australia	Pre/post-test	Multi-professional Learning Modules (MLMs)	Overall objectives (objectives for individual modules not provided): - For students to interact across professional boundaries - Give students opportunity to develop professional communication skills - Encourage students to explore boundaries of their own professional identity - For students to develop a better understanding of other health professions - Promote a healthcare work ethic based on effective teamwork	IPE	Two modules relevant to this review: ‘Self-Care and Child Protection’ 8 hours delivered in 3 sessions over 2 weeks ‘Child Protection in Clinical Practice’ 2 × 3 hour sessions delivered in a single day	*N* = 137 undergraduate students: - Nursing - Nutrition and dietetics - Medicine - Diagnostic radiography - Occupational therapy	Evaluation specific to module: **‘Self-care and child protection:’** “a multi-professional approach is very important in child protection” (5-point Likert scale). **‘Child Protection in Clinical Practice:’** Perceived importance of multi-professional approach on visual analog scale (10-points). Open-ended questions for both
Straub et al. (2017) Freiberg, Germany	Post-test	To help better—work together! interprofessional collaboration in child protection and family services in pediatrics	Course aims: - Distribution of knowledge and competencies regarding expertise - Approaches to problems of different health care professions - Identification of fostering and hindering factors for IPC - Common professional language and understanding - Reorganization of roles and limits of different health care professionals	IPE IPC IPL	2×, 4-hour in-person meetings 2 weeks apart. Self-study for 2 weeks	**Post-graduate** - Educational pedagogy - Social work - Psychology - Clinical pedagogy Undergraduate students Medical students	Self-reported quality of course and teaching 6-point Likert scale and open-ended questions. IPC and IPE: • Readiness for Interprofessional Learning Scale (RIPLS, 19 item) • Interprofessional self-assessment instrument (ISI, 21 items) • Modified ISI 27 items
Stuck et al. (2020) Hamburg, Kassel, Kiel, Merscburg & Munster, Germany	Pre/post-test	Sexualized Violence in Institutions	To teach students basic knowledge about sexualized violence in educational institutions, sexual socialization, and sex education	Not stated	Three seminars held over 14 weeks: - Sexualized violence in institutional settings - Sexual socialization and education - Professionalism and ethics	- Educational science - Social work - Social pedagogy Psychology	Self-report knowledge and confidence (6-point scale). Factual knowledge—24 questions on facts and terms. Belief in sex-related myths - validated questionnaires: *Acceptance of Modern Myths About Sexual Aggression Scale (AMMSA Scale)* *Child Sexual Abuse Myths Scale (CSAM Scale)*
Victor-Chmil et al. (2016) Philadelphia, USA	Post-test	Child Abuse Reporting Interprofessional Simulation-Based Learning Experience (CAR-ISBE)	To provide opportunities for students to - Be immersed in a realistic yet safe situation where CM is reported - Work together to problem solve - Collaborate to assess, provide care, and evaluate family dynamics in community setting	Experiential learning simulation model	Four phases—total 4 hours: thinking (3 hours online), planning, performing, and debriefing (20 minutes each, in-person)	*N* = 129 students - Post-graduate pharmacy (*n* = 74) - Undergraduate nursing (*n* = 55)	Self-report satisfaction and realism (Likert scale) student Open-ended questions (facilitator)
Whiteley et al. (2014), Gillespie et al. (2010) British Columbia, Canada	Post-test	Interprofessional Education (IPE) to Address Child Welfare: The Okanagan Project Or IPE in Child Welfare workshop (Whiteley et al., 2014)	Deepen understanding of own professional role in child welfare and promote understanding of other professions roles	- IPL - IPP - IPE - IPC - Modeling collaborative behavior - Problem based learning	Workshop (length not stated) with four scenario presentations followed by group discussions	Preservice - Nursing - Social work - Teacher education students	Self-report of usefulness, realism and changes to practice (Likert scale)

1 Knox L, ed. Youth violence and the health professions: core competencies for effective practice. Southern California Developing Center for Academic Excellence for Youth Violence Prevention, 2001. CM = child maltreatment; IPV = intimate partner violence; IPE = interprofessional education; IPC = interprofessional collaboration; IPL = interprofessional learning; IPP = interprofessional practice; FDV = family and domestic violence.

### Types of IPE

The type and focus of child maltreatment education was variable and had minimal consistency of its core content and scope. Some of the programs encompassed a holistic and broad approach to addressing child abuse and neglect through prevention, early intervention, and responses to instances of child maltreatment. For example, Almendingen et al. (2021) focused on children’s rights and socio-political and legal issues, and their associations with children’s health and development, advocating the importance of intersectoral support for children. Three studies focused primarily on prevention of maltreatment (Dolunayğ-Cuğ et al., 2022; Rodriguez-Frau & Mirabal-Colon, 2005; Sidelinger et al., 2005), while others (*n* = 2) emphasized identification, screening, and responses to suspected maltreatment (Johnson et al., 2022; Kuliukas et al., 2017).

Some programs only addressed a specific type of maltreatment such as sexual abuse (Stuck et al., 2020) or domestic and family violence (Kuliukas et al., 2017). Some studies reported educational interventions for IPE in child protection that were a subcomponent of a larger education program. For example, *Violence Across the Lifespan* was not specific to children, and covered human trafficking, child maltreatment and intimate partner violence (Johnson et al., 2022), while Smith et al. (2005) developed a five-module interprofessional skills program, of which only two were relevant to this review (*Self-Care and Child Protection*, and *Child Protection in Clinical Practice*).

**Figure 2.**
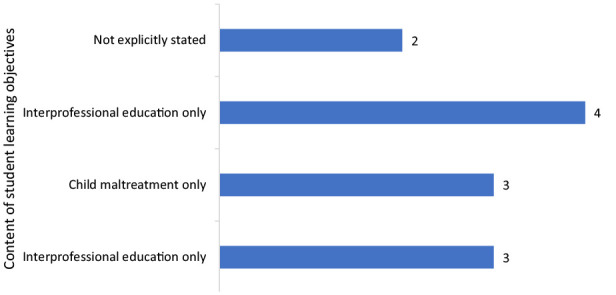
Focus of learning objectives of the educational interventions (*n* = 12).

The learning objectives of some educational interventions were not clearly stated and varied in their level of detail (see Figure 2). Some studies did not explicitly state learning objectives (Sidelinger et al., 2005) or learning objectives could only be implied by the aims/objectives of the research question (Almendingen et al., 2021). Alternatively, some studies provided one broad learning objective such as “*to teach students basic knowledge about sexualized violence in educational institutions, sexual socialization and sex education*” (Stuck et al., 2020, p. 701) or “*deepen understanding of one’s own professional role in child welfare while also promoting understanding of the different perspectives and roles of other professions*” (Whiteley et al., 2014, p. 151). In contrast, some studies had well-defined and detailed learning objectives which related to multiple aspects of child maltreatment (Dolunayğ-Cuğ et al., 2022; Rodriguez-Frau & Mirabal-Colon, 2005; Stuck et al., 2020), interprofessional practice (Smith et al., 2005; Straub et al., 2017; Whiteley et al., 2014), or both child maltreatment and interprofessional practice (Johnson, 2015; Johnson et al., 2022; Kuliukas et al., 2017; Victor-Chmil & Foote, 2016).

Although some (*n* = 4) educational programs incorporated student learning outcomes addressing both IPE and knowledge in child protection, others did not make interprofessional learning outcomes or components explicit. For example, Dolunayğ-Cuğ et al. (2022) a youth violence prevention program for law, health, and education students—focused on preventing and responding to child maltreatment without stating interprofessional learning goals. Stuck et al. (2020) and Sidelinger et al. (2005) were similar, emphasizing development of knowledge and skills about violence or abuse without explicitly indicating how interprofessional learning was incorporated. Similarly, Rodriguez-Frau and Mirabal-Colon (2005, p. 214) aimed to “train undergraduate health professional students in youth violence prevention” and expected that students would therefore interact with other disciplines—although it was unclear to what extent interactions occurred. The learning objectives for interprofessional practice reported by Rodriguez-Frau and Mirabal-Colon (2005) involved reflecting on health professional roles, commonalities/differences and importance of collaboration. Thus, although students were required to reflect on interprofessional roles, they did not necessarily engage in interprofessional learning or practice.

### Pedagogical and Conceptual Frameworks

There was no consistency of the pedagogical and conceptual frameworks that underpinned education for interprofessional practice in child protection for health and allied health professionals. Some manuscripts did not state any underpinning pedagogical or conceptual framework (Dolunayğ-Cuğ et al., 2022; Stuck et al., 2020) while others implied alignment with a specific framework through their overall narrative but did not explicitly apply it to the educational intervention (Johnson, 2015; Kuliukas et al., 2017). Seven of 12 educational interventions clearly articulated their pedagogical basis, which included IPE or practice (Almendingen et al., 2021; Smith et al., 2005; Straub et al., 2017; Whiteley et al., 2014), constructivist theory (Almendingen et al., 2021; Rodriguez-Frau & Mirabal-Colon, 2005), problem-based learning (Sidelinger et al., 2005), experiential learning (Sidelinger et al., 2005; Victor-Chmil & Foote, 2016), and modeling interprofessional collaboration (Whiteley et al., 2014). Three manuscripts identified principles that informed the child protection elements of educational content, and included Bronfenbrenner’s ecological system theory, United Nations Convention on the Rights of the Child 1989 and local legislation (Almendingen et al., 2021), trauma-informed principles (Johnson et al., 2022), and health professional competency standards (Rodriguez-Frau & Mirabal-Colon, 2005).

### Delivery of Education

Education was delivered in a diversity of ways and included combinations of online learning, classroom-based activities, simulations, and practical experiences with children or families. All but one study articulated classroom-based activities such as lectures, seminars, workshops, collaborative group work, case studies, role play, and/or clinical simulation. Sidelinger et al. (2005) described the implementation of a youth violence prevention educational intervention that was tailored differently across sites and did not report the specific learning activities. In some instances, classroom-based activities were supplemented by further online activities or materials (Almendingen et al., 2021; Johnson et al., 2022; Straub et al., 2017; Victor-Chmil & Foote, 2016). For example, interprofessional groups of students collaboratively worked through case studies relating to child protection supported with online learning resources (Almendingen et al., 2021; Straub et al., 2017; Victor-Chmil & Foote, 2016), or engaged in interprofessional discussion of topics such as child trafficking via an online discussion forum (Johnson et al., 2022).

Although educational interventions were reported as interprofessional, the details around how students were required to engage with students from other disciplines was not clearly reported. Some authors reported structured expectations for students to work interprofessionally during learning activities and/or assessments (Almendingen et al., 2021; Johnson et al., 2022; Kuliukas et al., 2017; Straub et al., 2017; Victor-Chmil & Foote, 2016; Whiteley et al., 2014), but the level of interprofessional interaction varied considerably. For example, Straub et al. (2017) required groups of 5 to 7 students from different disciplines to present a group poster of a child protection case study, while Johnson et al. (2022) explicitly required students to make and respond to online discussion forum posts with students from different disciplines. Thus, although students from different disciplines were enrolled in the courses, it was often unclear whether interprofessional learning was achieved.

Few (*n* = 4) educational interventions included practical experiences with children, young people or families, either through clinical simulation or placements. From these four, two used clinical simulations: a hi-fidelity simulated antenatal domestic violence situation with paid actors (Kuliukas et al., 2017) and a low-fidelity simulated caregiver opioid misuse scenario (Victor-Chmil & Foote, 2016). Practical experiences that placed students in direct contact with children, young people, and families were not clearly reported. Dolunayğ-Cuğ et al. (2022) offered students the opportunity for an 8-week practicum after completion of the theoretical component, but it is unclear how many students accepted this offer. In this example, Dolunayğ-Cuğ et al. (2022) had a practical experience that involved facilitation of a school-based violence prevention program for primary school students. Sidelinger et al. (2005) reported on multiple sites and indicated that one site had a 2-week community based experiential rotation—details not provided. Thus, educational interventions were varied and often underreported in how they were managed and received.

### Outcomes and Measures

Outcomes of educational intervention were frequently inadequately evaluated and/or reported with limited detail and description of the evaluations. Two programs had not yet been delivered and instead described the process of development, implementation, and/or informal evaluation (Johnson, 2015; Sidelinger et al., 2005). Studies that were formally evaluated mostly used post-test only design (*n* = 6) (Dolunayğ-Cuğ et al., 2022; Kuliukas et al., 2017; Rodriguez-Frau & Mirabal-Colon, 2005; Straub et al., 2017; Victor-Chmil & Foote, 2016; Whiteley et al., 2014) or pre/post-test (*n* = 4) (Almendingen et al., 2021; Johnson et al., 2022; Smith et al., 2005; Stuck et al., 2020). In these studies, students were invited to evaluate the program immediately after its completion, and in some instances, staff were also invited to participate in evaluations. Evaluation questions for students included Likert scales reporting satisfaction, realism of content, self-rating of knowledge, self-rating of collaborative skills, and open-ended responses. Only two studies used a pre-existing validated measure to evaluate student outcomes. Stuck et al. (2020) measured students’ belief in sex-related myths using the Acceptance of Modern Myths About Sexual Aggression Scale and Child Sexual Abuse Myths Scale. Straub et al. (2017) used a validated measure for interprofessional learning: Readiness for Interprofessional Learning Scale (RIPLS) and a measure developed by Straub et al.’s (2017) institution: Interprofessional Self-Assessment Instrument (ISI). Details of any validation of the ISI were not provided. Out of 10 studies that assessed students’ knowledge, attitudes and/or beliefs post-intervention, all measures occurred immediately after completion, with only one exception (Stuck et al., 2020) where follow-up also occurred at 6 months.

With limited application of validated measures, evaluations relied primarily upon student evaluations and self-reported learning which may not translate to greater knowledge and skills upon entry into professional practice. However, some authors described a lack of appropriate existing validated measures which could be used to evaluate the impact of education on students’ knowledge and skills (Almendingen et al., 2021; Smith et al., 2005).

### Academic Interest in Research on Scholarship

**Figure 3.**
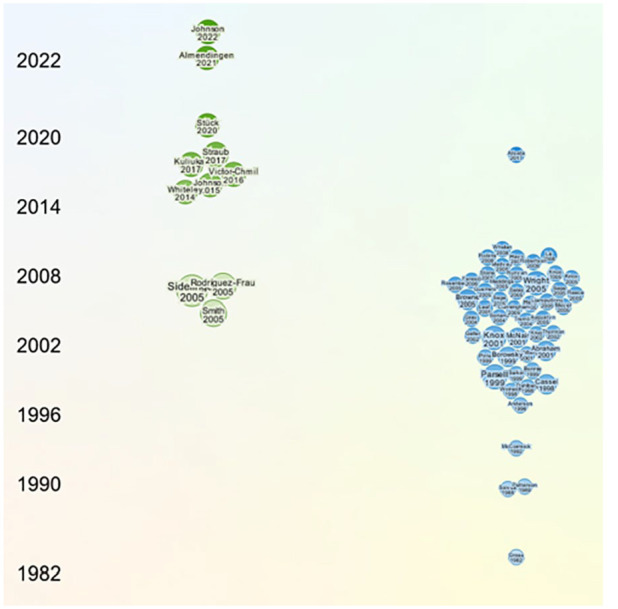
Timeline of selected papers and similar works.

The use of ResearchRabbit AI tool provided a timeline visualization for the 12 studies included (green), identifying 644 similar works (not shown) of which the 50 most relevant similar works (blue) are provided in Figure 3. While visualization indicated some growing interest in educational activity and publication related to interprofessional practice, mostly from 1996 to 2011 and peaking in 2008, the relevant similar works at best focus on post-graduate interprofessional development. It is possible that increased publication activity in interprofessional development in child maltreatment coincides with advocacy and subsequent rolling implementations of mandatory reporting laws across several countries (Mathews, 2015; McLaren, 2007). While the 12 studies included in the current review indicate some interest in taking interprofessional learning on child maltreatment into the classroom, corporatization and neo-liberalism in higher education has influenced academics to publish out of funded research projects as opposed to classroom activity (Martin-Sardesai et al., 2021; Zajda & Rust, 2020). It is most likely that preservice IPE in child maltreatment is simply underreported.

## Discussion

This scoping review explored the nature and scope of IPE for preservice health and allied health professionals in prevention, early intervention and responses for child maltreatment. Findings demonstrated that while IPE programs for preservice students exist, there was no consistency of target professions, learning objectives, underpinning frameworks, inclusion of cultural safety, or methods of delivery. Furthermore, there has been limited evaluation beyond pre/post student self-report of perceived impacts and satisfaction, with only two studies using validated questionnaires (Straub et al., 2017; Stuck et al., 2020) and one following up beyond the initial post-intervention period (Stuck et al., 2020). Reporting of methods and findings were generally low quality, and none of the interventions has been replicated beyond its original context.

Although there is a great need for education that effectively prepares preservice health and allied health professionals for interprofessional practice in prevention and responses to child maltreatment, none of the educational programs comprehensively addressed both interprofessional practice and child maltreatment. Many studies did not define interprofessional practice or articulate explicit interprofessional learning objectives, which raises questions about the quality and comprehensiveness of how interprofessional was included (Rogers et al., 2017). A lack of consistent underpinning interprofessional frameworks a common criticism of IPE and even when IPE is clearly articulated it can be “tokenistic and piecemeal” rather than showing meaningful interprofessional learning has occurred (Bogossian & Craven, 2021; Shakhovskoy et al., 2022). In short, simply undertaking education with students from other disciplines does not necessarily achieve interprofessional learning if interprofessional learning outcomes are not made explicit (Bogossian et al., 2023).

Findings highlighted the importance of differentiating between the World Health Organization (2010, p. 7) definition where students from “two or more professions learn about, from and with each other” versus interdisciplinary approaches where curriculum does not explicitly incorporate interprofessional learning opportunities. Some studies within this review explicitly incorporated interprofessional learning by requiring interprofessional student groups to collaborate on a learning task (Kuliukas et al., 2017; Smith et al., 2005), but other studies did not state how or if students were required to interact across disciplinary boundaries (Dolunayğ-Cuğ et al., 2022; Rodriguez-Frau & Mirabal-Colon, 2005; Sidelinger et al., 2005; Stuck et al., 2020). Similar variability in fidelity of interprofessional learning is present in other major child maltreatment education and training programs. For example, Child Advocacy Studies Training (CAST) is widely implemented across the USA as “courses and certificate or minor programs to educate undergraduate and graduate students about child maltreatment” (Vieth et al., 2019). Although CAST curriculum was developed by a multidisciplinary team for delivery to all professions working with children, IPE is not a core component of CAST which is also delivered in uni-professional contexts such as medicine or law (Knox & Pelletier, 2014; Vieth et al., 2019).

Furthermore, most examples of interprofessional child maltreatment content related to very specific aspects of child maltreatment (i.e., mandatory reporting, youth violence, sexualized violence, domestic, and family violence), and therefore did not represent the full range of knowledge and skills required to effectively address child maltreatment. Only one study (Almendingen et al., 2021) reported a broader focus on children’s rights, although did not explicitly emphasize development of competency across the spectrum of prevention, early intervention and responses to maltreatment. As outlined by Lonne et al. (2020), a public health response to child maltreatment requires reorienting the crisis-focused child and family workforce with knowledge, skills and models for supporting children and families across a continuum of service provision including prevention and early intervention. A public health approach also recognizes how socio-economic conditions can lead to higher burdens of child maltreatment in specific population groups. For example, First Nations populations are more likely to experience child protection intervention due to impacts of colonization, systemic racism, punitive social policies, intergenerational trauma and loss of land, cultural knowledge, and kinship structures (United Nations, 2021; World Health Organization, 2022b). Partnering with population groups most impacted by child maltreatment is essential to inform effective support inclusive of culturally safe care provisions for their often complex and nuanced needs. For example, Reid et al. (2022) highlighted the importance of tailoring perinatal support to the specific cultural and relational needs of First Nations parents who had experienced complex trauma. Further research should explore how knowledge and skills for effective interprofessional support for priority populations can be integrated into future curriculum.

More broadly, many educational programs exist to enhance reporting of child maltreatment for qualified professionals (Walsh et al., 2022) or develop interprofessional competency (Bogossian et al., 2023). However, with a shift toward a public health approach, the workforce needs preparation for both collaborative skills and skills for prevention and early intervention of child maltreatment (Lonne et al., 2020; Russ et al., 2022). Learning needs will be unique for preservice students due to novice knowledge and skills in interprofessional practice and child health and development. For example, when developing skills for interprofessional practice, undergraduate professionals first need to develop an understanding of their own professional identity and role to inform interactions with other professionals (Shakhovskoy et al., 2022; Teodorczuk et al., 2016). Similarly, preservice health and allied heath students will have limited knowledge of the principles underpinning prevention and response to child maltreatment, including child health and development, and models for working with children/families.

There are many challenges associated with developing, delivering, and coordinating interprofessional child protection education, including competing priorities and schedules across disciplines and addressing requirements of professional accreditation bodies (Kuliukas et al., 2017; Straub et al., 2017; Victor-Chmil & Foote, 2016; Whiteley et al., 2014). These challenges were not unique to child protection, with many authors in other contexts exploring IPE reporting these same difficulties (Fox et al., 2018; Teodorczuk et al., 2016). Consequently, delivery and coordination of preservice IPE for health and allied health professionals will require transformational leadership within educational institutions (Bogossian et al., 2023) to address challenges that arise during development, planning, and implementation. For example, Packard et al. (2018) addressed challenges of implementing interprofessional curriculum into a multi-campus university through a core interprofessional leadership team that facilitated stakeholder engagement, promoted grassroots initiatives, developed dedicated institutional support structures, and created opportunities for IPE innovation from all staff. Furthermore, there are unique challenges to delivering child protection education including its emotive nature, inherent complexity, and need to develop advanced critical reflective skills (Egonsdotter et al., 2020; Keys, 2016), further adding to the complexities of integrating interprofessional child protection education into preservice curriculum.

## Limitations

This is the first review to systematically synthesize current evidence for health and allied health professional IPE for child protection, and findings should be interpreted with acknowledgment of the following limitations. Studies were limited to English language studies and thus may have missed relevant studies published in other languages. Our search strategy was robust, but it is possible that educational interventions published in non-traditional mediums or in other gray literature are not represented, although we consider this unlikely because no such examples were identified through checking of manuscript reference lists. It is possible that our results may not be generalizable to other contexts, such as non-English speaking countries and future searches could be expanded to non-English language studies and reports within the gray literature. Furthermore, a small number of studies did not clearly define interprofessional practice/education or articulate how interprofessional elements were embedded throughout their educational program. Thus, it is possible that some studies that claimed to be interprofessional, did not meet the accepted definition of IPE where “two or more professions learn about, from and with each other” (World Health Organization, 2010).

## Conclusion

There is little published research exploring IPE for preservice health and allied health professionals in child protection. Furthermore, research that does exist is poorly reported and lacks the methodological quality to make recommendations for practice. Thus, there is an urgent need to develop and rigorously evaluate educational curricula if professionals are to effectively work together to prevent and respond to child maltreatment. In the absence of clear interprofessional learning shared across health and allied health disciplines, poor collaboration for children at risk of maltreatment is likely to continue with dire consequences. Importantly, future educational interventions need to both incorporate the range of skills for a comprehensive public health response to child maltreatment and explicitly articulate how IPE is achieved. Although many challenges will present during the stages of development, evaluation, and implementation, effective IPE in child maltreatment is an important part of global efforts to improve collaborative responses to children and families experiencing adversities. Further work should explore development of standardized interprofessional learning objectives across professions and ensure educational interventions are robustly evaluated and replicated across diverse contexts.

**Table:** Summary Table—Critical Findings.

Critical findings of the review
Interprofessional child protection education for health and allied health professionals is poorly reported and lacking robust evaluation
Learning objectives were often inconsistent or missing
Interprofessional learning objectives and methods for achieving interprofessional learning were often missing
Child maltreatment content was often incomplete, focusing on specific responses or types of maltreatment
Implications of the review for practice, policy, and research
There is currently no evidence base to inform comprehensive and effective interprofessional child protection education for health and allied health professionals
Development of consistent educational interventions that comprehensively address interprofessional and child maltreatment content are needed
Development of these educational interventions needs to be robustly evaluated to assess impact on professional knowledge and collaborative skills

## Supplemental Material

sj-docx-1-tva-10.1177_15248380231221279 – Supplemental material for Interprofessional Education in Child Protection for Preservice Health and Allied Health Professionals: A Scoping Review Supplemental material, sj-docx-1-tva-10.1177_15248380231221279 for Interprofessional Education in Child Protection for Preservice Health and Allied Health Professionals: A Scoping Review by Lauren Elizabeth Lines, Tracy Alexis Kakyo, Helen McLaren, Megan Cooper, Nina Sivertsen, Alison Hutton, Lana Zannettino, Rebecca Starrs, Donna Hartz, Shannon Brown and Julian Grant in Trauma, Violence, & Abuse
