# Supplementary material for: Interprofessional Education in Child Protection for Preservice Health and Allied Health Professionals: A Scoping Review
Source: Trauma Violence Abuse. 2024 Jan 28;25(4):2657–71. doi: 10.1177/15248380231221279 (PMC11370204; doi:10.1177/15248380231221279)
Supplement: sj-docx-1-tva-10.1177_15248380231221279 – Supplemental material for Interprofessional Education in Child Protection for Preservice Health and Allied Health Professionals: A Scoping Review [file sj-docx-1-tva-10.1177_15248380231221279.docx]

1. **Supplementary Online File One: Search Strategy and results for Medline**

| Ovid MEDLINE(R) and Epub Ahead of Print, In-Process, In-Data-Review & Other Non-Indexed Citations, Daily and Versions <1946 to February 06, 2023> | | |
| --- | --- | --- |
| 1 | domestic violence/ or child abuse/ or child abuse, sexual/ or intimate partner violence/ or spouse abuse/ or Child Welfare/ | 71205 |
| 2 | (incest/ or sex work/ or sex offenses/ or human trafficking/ or rape/) and (baby or babies or newborn* infant* or toddler* or child or children or minor or minors or "young people" or youth* or adolescen* or "young person*" or teen* or juvenile*).tw,kf. | 5431 |
| 3 | ((female* or wife or wives or husband* or child or children or minors or spouse or spousal or domestic) adj3 (battery or battered)).tw,kf. | 2145 |
| 4 | ((baby or babies or newborn* infant* or toddler* or child or children or minor or minors or "young people" or youth* or adolescen* or "young person*" or teen* or juvenile*) adj4 (welfare or "protective services" or protection or safeguard* or abus* or neglect* or maltreat* or mistreat or exploit*)).tw,kf. | 39586 |
| 5 | ((baby or babies or newborn* infant* or toddler* or child or children or minor or minors or "young people" or youth* or adolescen* or "young person*" or teen* or juvenile*) and (incest* or "survival sex" or incest* or (transaction* adj4 sex*) or "sex traffic*" or prostitution or rape* or "sex work*" or "sex offen*" or "trading sex*" or "selling sex*" or porn or pornography or "Sex industry" or (sex adj4 swap*) or (sex* adj4 exchange*))).tw,id. | 5154 |
| 6 | ((domestic or family or partner or spousal or family or baby or babies or newborn* infant* or toddler* or child or children or minor or minors or "young people" or youth* or adolescen* or "young person*" or teen* or juvenile*) adj3 (violence or abuse or assault*)).tw,kf. | 44193 |
| 7 | or/1-6 | 103837 |
| 8 | education, medical, undergraduate/ or education, nursing, associate/ or education, nursing, baccalaureate/ or education, nursing, diploma programs/ | 51284 |
| 9 | Interdisciplinary Studies/ | 1246 |
| 10 | 8 and 9 | 34 |
| 11 | (("cross-disciplin*" or interdisciplinary or "inter-disciplinary" or multidisciplinary or "multi-disciplinary" or "inter-profession*" or "care team*") adj6 (Undergraduate* or college or preregistration or "pre-registration" or "pre-service" or preservice or baccalaureate or educat* or "study program*" or curriculum or pedagogy or student* or tutorial*)).tw,kf. | 6494 |
| 12 | 10 or 11 | 6516 |
| 13 | 7 and 12 | 65 |

**Medline Guide:**

**/** = Subject Heading or MeSH heading

1. * = Truncation symbol, will find all variations of the words’ ending – eg **therap*** will find **therapist, therapists, therapy, therapies**
2. **.ti,ab.** = searching for these words only in the title or abstract of the paper
3. **.kf.** = searching for these words in the author supplied keywords
4. **adj3** = Adjacency – words need to be within 3 (can be a different number) words of each other
5. **.pt.** = Publication type
6. **or/x-y** = Boolean search of OR – used to combine all variations of words within one concept, will make the results bigger
7. **and/x,y,z** = Boolean search of AND – will tie the concepts together and make the results smaller
